# P25 TB or not TB? An unusual case of inflammatory arthritis in an anti-CCP positive patient

**DOI:** 10.1093/rap/rkad070.046

**Published:** 2023-09-27

**Authors:** Mariam Al-Attar, Sonia Shrimanker, William Dixon

**Affiliations:** Salford Royal Hospital, Northern Care Alliance NHS Foundation Trust, Salford, United Kingdom; Centre for Epidemiology Versus Arthritis, University of Manchester, Manchester, United Kingdom; Salford Royal Hospital, Northern Care Alliance NHS Foundation Trust, Salford, United Kingdom; Salford Royal Hospital, Northern Care Alliance NHS Foundation Trust, Salford, United Kingdom; Centre for Epidemiology Versus Arthritis, University of Manchester, Manchester, United Kingdom

## Abstract

**Introduction:**

Rheumatoid arthritis (RA) is the most common inflammatory arthritis, typically presenting with a symmetrical small joint polyarthritis. It has been classically associated with rheumatoid factor (RF) positivity and, more recently, anti-cyclic citrullinated peptide (CCP) positivity which has shown greater sensitivity and specificity. Wider differential diagnoses should always be considered in a patient presenting with inflammatory arthritis, even if the antibody profile is suggestive of RA. This case highlights the importance of re-evaluating an RA diagnosis if the clinical course is atypical. The relevance of anti-CCP antibodies will also be explored in relation to this case and beyond.

**Case description:**

A 41-year-old female who had recently moved to the United Kingdom from Pakistan was seen as a new referral to the rheumatology clinic. She had a medical history of RA diagnosed in Pakistan and was currently on antibiotics for a dental abscess. She complained of right wrist pain, swelling and significant morning stiffness. She described intermittent PIP and right knee pain. On examination her right wrist was tender and swollen. Her right middle MCP was tender, but not swollen. Her right knee was tender with crepitus and a mild effusion, and she had a mildly tender left MTP squeeze. She had been taking methotrexate, leflunomide, hydroxychloroquine and 10mg prednisolone for 6 years.

Given her dental infection, she was advised to pause immunosuppressive therapy. X-rays were requested along with baseline bloods, autoantibodies and pre-biologic screening, pre-empting the need to escalate treatment given active disease on triple disease-modifying anti-rheumatic drug (DMARD) therapy.

One week later, results revealed positive RF (154 IU/ml) and anti-CCP (120 units/ml) as expected, along with an unexpected positive QuantiFERON test. She was evaluated in tuberculosis (TB) clinic and treated for latent TB. When followed up in rheumatology clinic, she had been off DMARD therapy and on isoniazid treatment for several months. Surprisingly, her wrist pain and swelling had resolved, and she had no tender or swollen joints. Review of X-rays revealed loss of joint space at inter-, radio- and ulnar-carpal joints, widespread erosions and subchondral cysts to the right wrist only, with a normal left wrist X-Ray. The combination of clinical improvement off DMARD therapy and asymmetrical joint damage prompted consideration that this may not be RA. A diagnosis of TB arthritis was considered likely at this point, and she has been referred for synovial biopsy of the right wrist, which is scheduled to occur soon.

**Discussion:**

We have presented the case of a patient with a suspected misdiagnosis of RA. Although her diagnosis has not yet been confirmed, this case still raises important learning points around anti-CCP antibodies and musculoskeletal manifestations of TB as a differential diagnosis for inflammatory arthritis.

Studies have indicated that systemic autoimmunity, as evidenced by positive anti-CCP antibodies, may be detectable up to 10 years before the development of clinically evident RA. This phase has been labelled by some as ‘pre-clinical RA’, particularly when there are associated symptoms such as arthralgia or fatigue; however, some will never go on to develop clinical RA. In our patient who had evidence of inflammatory arthritis atypical for RA, the significance of her anti-CCP positivity is unclear. If her synovial biopsy confirms TB arthritis, this does not negate a concomitant diagnosis of RA, nor the development of RA in the future. Her clinical course will therefore need close monitoring. Some reports suggest that TB can induce anti-CCP positivity, but it remains unclear whether this constitutes a ‘false-positivity’, or whether it confers a future risk of RA.

QuantiFERON screening was not, in this case, intended as a diagnostic test and, when assessed in TB clinic, her joint symptoms were not considered as a potential TB manifestation. This case serves as a reminder to consider musculoskeletal TB manifestations (around 10% of extrapulmonary TB) as a differential diagnosis to RA in at-risk populations presenting with inflammatory arthritis, particularly in immunosuppressed patients and those who have lived in TB-endemic areas. TB arthritis most commonly manifests as a monoarthritis of weight-bearing joints, but diagnosis is often delayed due to varied clinical presentation and radiological appearances. The gold standard diagnostic test is synovial biopsy, demonstrating characteristic caseating granulomas. Early antimicrobial therapy results in near-complete resolution and preservation of joint function.

**Key learning points:**

This case report highlights the importance of maintaining a broad differential diagnosis for inflammatory arthritis. With an increasingly diverse population accessing healthcare in the United Kingdom, clinicians should be vigilant for atypical presentations and consider screening for TB in individuals with relevant risk factors. Although QuantiFERON testing is mandated as a pre-biologic screen due to the specific role of TNF-α in latent TB compartmentalisation, we feel there may be a benefit to extending this screening to DMARD therapies in at-risk populations. In our patient, it is not known whether she developed TB whilst in an immunocompromised state on DMARD therapy, alongside an existing RA diagnosis, or whether her initial presentation was of TB arthritis misdiagnosed as RA. In any case, given the immunosuppressive nature of DMARD therapies, we believe a cautious screening approach would be beneficial.

A further learning point is regarding the re-evaluation of previous diagnoses, particularly if faced with an incongruent clinical picture. A previously labelled diagnosis can introduce subconscious cognitive bias, risking a failure to approach the clinical scenario objectively. This case emphasises the importance of critical thinking and thorough clinical assessments, remembering that positive anti-CCP or RF antibodies do not equate to an RA diagnosis.

In summary, this case serves as a reminder for rheumatologists to approach each patient with an open mind and to maintain critical thought processes, adapting and accounting for individual patient characteristics and risk factors. Our key learning objective from attending the case-based conference will be to broaden our exposure to unusual and atypical cases of RA, in order to improve our clinical acumen and ensure that we provide the best possible care to patients presenting with inflammatory arthritis.

